# Combining Virtual Reality and Biofeedback to Foster Empathic Abilities in Humans

**DOI:** 10.3389/fpsyg.2018.02741

**Published:** 2019-02-05

**Authors:** Felix Schoeller, Philippe Bertrand, Lynda Joy Gerry, Abhinandan Jain, Adam Haar Horowitz, Franck Zenasni

**Affiliations:** ^1^Centre de Recherches Interdisciplinaires, Université Paris Descartes, Paris, France; ^2^U1001, Institut National de la santé et de la Recherche Médicale, Paris, France; ^3^VR Frontiers Lab (CRI Labs), Institut Innovant de Formation par la Recherche, USPC, Centre de Recherches Interdisciplinaires, Paris, France; ^4^Laboratoire de Psychologie et d'Ergonomie Appliquées (UMR), Université Paris Descartes - Sorbonne Paris Cité, Institut de Psychologie, Paris, France; ^5^BeAnotherLab Research, BeAnotherLab Association, Barcelona, Spain; ^6^Multisensory Experience Lab, Aalborg University Copenhagen, Copenhagen, Denmark; ^7^Enactive Virtuality Lab, Tallinn University, Tallinn, Estonia; ^8^Fluid Interfaces Group, MIT Media Lab, Cambridge, MA, United States

**Keywords:** empathy, compassion, aesthetic chills, psychogenic shivers, altruism, technology, biofeedback, emotion-regulation

Recent technological advances coupled with progress in brain and psychological sciences allow the controlled induction and regulation of human psychophysiological states. These progresses often aim toward the goal of developing human-machine interfaces to improve human factors such as mental health, human relations, well-being, and empathy. In this short article, we present some of such devices with a particular emphasis on technology aiming to foster empathic abilities in humans; that is, our ability to care, understand, and help our fellow human beings. In order to discuss any possible use for such devices in a clinical setting, we start by outlining definitions for the terms used in the article, and present three devices designed with the goal of modulating empathy in humans.

## Understanding Empathic Phenomena

Empathy is a vague term often used to describe a broad range of phenomena. These empathic phenomena are related to several interconnected motor, cognitive, emotional, motivational, and behavioral functions (McCall and Singer, 2013). Two of the most discussed aspects of empathy are affective and cognitive empathy. Cognitive empathy simply refers to the ability to predict various mental states of another person (Reniers et al., 2011; Myszkowski et al., 2017). Affective empathy refers to an isomorphic emotional response to somebody else's feelings, combined with a clear self-other distinction—i.e., a metacognitive ability to recognize another as being separate from oneself (de Vignemont and Singer, 2006; Decety and Meyer, 2008; Singer and Lamm, 2009; Decety, 2010).

Affective empathy may lead to both positive and negative effects. Highly distressing empathic stimuli for example can lead observers to develop empathic distress, which in turn may lead to burnout (Hojat et al., 2009; Klimecki et al., 2013), or to self-centered responses with negative consequences on social behavior (Decety, 2010). To a certain extent, the negative effects of affective empathy can be trained, limited, and avoided. When such top-down processes of emotion regulation are successful in limiting the stress response, subjects can learn to transform empathic distress into healthy and prosocial reactions.

One critical prosocial mechanism is the emotion of compassion (Decety, 2010). Compassion involves recognizing someone else as part of a collective shared humanity and extending benevolence and care for the welfare of that individual. While empathy triggers a parallel isomorphic emotion to another person's distress, compassion has been found to trigger neural areas related to affiliation (Klimecki et al., 2014). In the following sections we examine how various technologies attempt to predict and control both negative and positive effects of these empathic phenomena at the three levels of mind, brain, and behavior.

## Biofeedback and Brain Control Interfaces to Modulate Empathy

Biofeedback mechanisms and brain-control interfaces are two of the main kinds of technology aiming to regulate the negative effects of affective empathy. Bio-feedback mechanisms are interactive systems, which record biosensor data to provide real-time feedback to the user through data visualizations, games, cinematic narratives, or virtual environments. Biofeedback systems have been incorporated into so called “serious games” to train self-awareness and emotion regulation abilities. This is often done so by means of reward: better performance at emotion regulation leads to in-game advantages.

For example, feedback of cardiovascular related signals has been found effective in reducing anxiety before public speaking presentations (Azevedo et al., 2017). By decreasing heart rate through a tactile feedback device, Azevedo and colleagues led participants to experience relaxation in a stressful context, in comparison to subjects exposed to accurate biofeedback. Concerning affective empathy and social emotions, one biofeedback game created by Blandon et al. (2016) aims at enhancing empathy in teenage populations. In this game, virtual telekinetic abilities and time-manipulation are mapped to changes in attention and self-regulation. In order to effectively cooperate and solve tasks collaboratively, the user must control attention and stress in response to visual parameters and objects.

Another line of work aiming to regulate affective empathy are brain computer interfaces (BCI). BCI function by measuring brain activity, extracting neural signatures, translating such neural signatures into computer commands, and providing this information back to the users through an interface (Vourvopoulos et al., 2017). BCI use neural measures to index attentional, emotional, and cognitive states. For example, evidence suggests that frontal alpha asymmetry can provide reliable estimation of emotional valence (Davidson et al., 1990), and that prefrontal brain activity is strongly related to the process of mentalizing (Heatherton et al., 2006). These studies are conducted under the assumption that users provided with information about their own psychophysiological activity through BCI feedback become increasingly aware of their own empathic arousal and cognitive engagement (Cavazza et al., 2014). Some researchers have also argued that combining biofeedback with compassion practices can maximize psychological and physical effects (e.g., Klich, 2015). Compassion meditation training is a technique for improving emotional self-regulation (Pace et al., 2009), and previous research has demonstrated effects of compassion training techniques on decreased stress-related behavioral and neurobiological responses (Carson et al., 2005; Gilbert and Procter, 2006; Lutz et al., 2008).

This early research is promising, yet much work needs to be conducted to verify the effects of technologies such as biofeedback and BCI on the regulation of human empathy. The following section presents a more specific technique to train empathic abilities, where users are invited to experience the sensory input of another person rather than their own. Embodied Virtual Reality (EVR), allows users to access sensorial data concerning the body of another person, its immediate context and peripersonal space.

## Embodied Virtual Reality for Empathy Training

The illusion of body ownership, or embodiment illusion, is a technique allowing users to virtually experience the body of another person (Botvinick and Cohen, 1998; Maselli and Slater, 2013). This alteration of self-representation through virtual reality (EVR) may lead to changes in behavior, referred to as *the proteus effect* (Yee and Bailenson, 2007). It seems that this effect relies much on the user's social schemas, stereotypes and models. For example, male subjects embodying an avatar of Sigmund Freud dialoguing about their personal problems with a virtual self can improve mood (Osimo et al., 2015). Similarly, embodying an avatar of Albert Einstein may increase performances in cognitive tasks (Banakou et al., 2018). By combining multimodal sensory and motor stimulations with first-person perspective visual input, this type of experience can lead users to feel actively engaged in a controlled virtual environment, and to behave in a realistic manner, adopting new attitudes, modulating cognitive biases and behavioral responses (Bertrand et al., 2018).

EVR have shown promising effects on increasing altruistic intentions and self-compassion (Rosenberg et al., 2013; Falconer et al., 2016). It has also proved useful in reducing short term implicit racial bias (Peck et al., 2013; Banakou et al., 2016). Recent experiments have shown that EVR can be used to alter behavior of aggressive populations (Seinfeld et al., 2018). After experiencing the perspective of a female victim of domestic abuses, offenders developed higher ability to recognize fearful female faces, reducing their bias of identifying fearful female faces of happy. Combined to biofeedback mechanisms these may offer powerful tool to study and modulate empathy in a clinical setting.

One of such embodiment systems is The Machine To Be Another (TMBA). This EVR has been widely presented in various artistic venues and uses different setups to allow two persons to exchange bodies in real time (Bertrand et al., 2014, 2018; Sutherland, 2014; Oliveira et al., 2016), and to allow users to experience the perspective and personal narrative of another human subject (Souppouris, 2014; Martín, 2016; Aragão, 2017). By allowing the use of real human individuals as characters to be embodied, TMBA has been applied to address issues in several contexts of prejudices, allowing a wide variety of users to experience the daily lives of migrants in Europe, muslims suffering prejudice, exiled individuals seeking asylum, and victims of military forces. TMBA has been used as a tool to bridge the gap between cultures, ideologies and backgrounds, aiming to expand individual's empathy toward actual human beings often considered outgroups, and to foster compassion (see Bertrand et al., 2018).

The current democratization of VR due to a greater access in technology allows the development of highly scalable therapeutic and educational methods for helping subjects develop positive emotional states and pro-social behavior. In the final section we present a proof of concept of how such EVR could serve to trigger more specific social emotions and their psychophysiological effects on mind and behavior in clinical and typical populations.

## Sensor-Actuator System to Control Social Emotions

Technologists attempting to control emotions in human populations to foster altruism can either study the needs and conflicts of such populations, or target specific social emotions and their effect on human altruism. One of such social emotions is aesthetic chills or psychogenic shivers (goosebumps, gooseflesh, chills, thrills). In this section, we review the devices attempting to control this emotion and its psychological effects in humans.

Psychogenic shivering (PS) has received considerable attention due to the fact that it is conscious, measurable and universal (Pelowski et al., 2018; Schoeller et al., 2018b). Shivers are a small muscle tremor ordinarily involved in the regulation of body temperature (Haman and Blondin, 2017). Yet humans sometimes shiver independently of any changes in temperature (Schoeller, 2015a). Here, we refer to this event as psychogenic shivering (PS). Psychogenic shivers are most often triggered by music and film, but can also occur in the course of scientific or religious practices (Schoeller, 2015b). What is of particular interest to this article is the fact that humans often shiver in reaction to specific social situations (Schurtz et al., 2011; Schoeller et al., 2018b) and that PS inducing music has been found to enhance altruism (Fukui and Toyoshima, 2014). It is interesting to note that other primates (most notably chimpanzees) also show signs of psychogenic shivering in the course of bluff display (Aureli and de Waal, 1977). One striking property of situations provoking PS in humans is their social dimension (e.g., Keltner and Haidt, 2003). Humans shiver in groups, specifically when the group shares a common goal and when each member of the group focuses its attention on the evolution of context in light of the goal (in a football stadium for example, or in a public manifestation). PS can be elicited by music (Blood and Zatorre, 2001), films (Schoeller and Perlovsky, 2016), science (Cronin and Greenwood, 1982; Schoeller, 2015b), religious ceremonies (Inbody, 2015; Schoeller, 2015b), social situations. Interestingly, in all these domains, negative PS related to fear and lack of information can be observed as well (Halpern et al., 1986; Zald and Pardo, 2002).

In past decades various technological projects have been involved in the control of PS. Teams have been interested in measuring (Kim et al., 2014), controlling (Jain, Horowitz and Schoeller), or incorporating PS in VR (Neidlinger K. et al., 2017; Neidlinger K. L. et al., 2017; Quesnel and Riecke, 2017, 2018; Quesnel et al., 2018). An important questions, currently beyond measurement, is whether the amount of heat related to specific emotions can be predicted effectively (Schoeller et al., 2018a), that is whether the psychogenic shivers serves any evolutionary function (Tihanyi et al., 2018). Progress toward answering this question could help better understand the neural networks underlying human social emotions and their biological importance in humans (Dunbar, 2009).

There have been several attempts to control the generation of PS through actuators (e.g., Neidlinger K. et al., 2017; Neidlinger K. L. et al., 2017). An ongoing collaboration amongst the authors involves the creation of a wearable chill-actuator device designed to create artificial chills and shivers. Currently, the device form factor includes a custom circuit board, a vibrating motor disc, and a peltier thermoelectric cooler housed in a body-fitted photopolymer resin case. This form factor allows for concurrent delivery of sudden cooling temperature and concentrated vibration on the upper back of participants, where organic PS typically begin, to initiate artificial shivers. The device is tied to a custom Android application and can be triggered wirelessly and remotely, allowing for single or multiple networked devices to deliver chills at predefined moments during songs or videos which subjects are viewing.

Past research has elucidated the correlations between PS and many metrics relevant to the generation of empathy, including levels of openness to experience, awe, and perceived meaningfulness of a situation (e.g., McCrae, 2007; Schurtz et al., 2011; Fayn et al., 2017; Quesnel et al., 2018; Schoeller et al., 2018b). Devices such as the PS actuator thus allow future testing of whether these correlational relationships previously seen are also causal, allowing for the generation of greater openness to experience through generation of artificially induced chills (Schoeller and Perlovsky, 2015). If validated, this would allow researchers to induce and maximize social emotions through bodily actuators at a distance.

## Conclusion

Different technological approaches, from biofeedback signals to embodied virtual reality and psychophysiological actuators, have been used to support the enhancement of human empathic abilities. The complementary nature of these stimuli regarding the different effects they may trigger lead us to reflect on the possible combination of these technologies to produce even more effective responses. For example, heart-rate biofeedback has been shown effective in enhancing embodiment (Aspell et al., 2013; Suzuki et al., 2013), but as previously discussed could also be used to enhance emotion regulation, an important aspect of avoiding empathic distress. One can envision a system combining both stimuli to help users to step into the shoes of a distressed individual while training for compassion. In the same way, VR could be used to offer immersive neurofeedback environments and help develop fine grained awareness of one's own empathic arousal and cognitive engagement. VR and PS could be combined to create social situations and shared goals for networked users in immersive environments.

In order to increase a sense of shared goals in VR, a chill-actuator device could be incorporated in such situations, increasing the likelihood of PS and theoretically increasing correlated social emotions such as shared group identity or synchronicity. The design of chill-eliciting VR stimuli, and the incorporation of chill-actuator devices into existing empathy related VR experiences such as TMBA, offer unique avenues for the study and generation of empathy and the physiological underpinnings of social emotions. Although further research is required to properly explore the effects of integrating these technologies, the development of new systems of empathic abilities reveals to be a promising field for positive technologies.

## Author Contributions

All authors listed have made a substantial, direct and intellectual contribution to the work, and approved it for publication.

### Conflict of Interest Statement

PB is a cocreator of one the art/research works mentioned in the article—The Machine to Be Another—and cofounder of the non-profit cultural association BeAnotherLab that presents this work in several contexts. The remaining authors declare that the research was conducted in the absence of any commercial or financial relationships that could be construed as a potential conflict of interest.
